# Immune checkpoint inhibitors in infectious disease

**DOI:** 10.1111/imr.13388

**Published:** 2024-09-09

**Authors:** Hannah A. D. King, Sharon R. Lewin

**Affiliations:** ^1^ Department of Infectious Diseases The University of Melbourne at The Peter Doherty Institute for Infection and Immunity Melbourne Victoria Australia; ^2^ Victorian Infectious Diseases Service Royal Melbourne Hospital at The Peter Doherty Institute for Infection and Immunity Melbourne Victoria Australia; ^3^ Department of Infectious Diseases Alfred Hospital and Monash University Melbourne Victoria Australia

**Keywords:** hepatitis B, HIV, immune checkpoint, immune checkpoint inhibitors, PD‐1

## Abstract

Following success in cancer immunotherapy, immune checkpoint blockade is emerging as an exciting potential treatment for some infectious diseases, specifically two chronic viral infections, HIV and hepatitis B. Here, we will discuss the function of immune checkpoints, their role in infectious disease pathology, and the ability of immune checkpoint blockade to reinvigorate the immune response. We focus on blockade of programmed cell death 1 (PD‐1) to induce durable immune‐mediated control of HIV, given that anti‐PD‐1 can restore function to exhausted HIV‐specific T cells and also reverse HIV latency, a long‐lived form of viral infection. We highlight several key studies and future directions of research in relation to anti‐PD‐1 and HIV persistence from our group, including the impact of immune checkpoint blockade on the establishment (*AIDS*, 2018, 32, 1491), maintenance (*PLoS Pathog*, 2016, 12, e1005761; *J Infect Dis*, 2017, 215, 911; *Cell Rep Med*, 2022, 3, 100766) and reversal of HIV latency (*Nat Commun*, 2019, 10, 814; *J Immunol*, 2020, 204, 1242), enhancement of HIV‐specific T cell function (*J Immunol*, 2022, 208, 54; *iScience*, 2023, 26, 108165), and investigating the effects of anti‐PD‐1 and anti‐CTLA‐4 *in vivo* in people with HIV on ART with cancer (*Sci Transl Med*, 2022, 14, eabl3836; *AIDS*, 2021, 35, 1631; *Clin Infect Dis*, 2021, 73, e1973). Our future work will focus on the impact of anti‐PD‐1 *in vivo* in people with HIV on ART without cancer and potential combinations of anti‐PD‐1 with other interventions, including therapeutic vaccines or antibodies and less toxic immune checkpoint blockers.

## 1 IMMUNE CHECKPOINT MOLECULES

Immune checkpoints are regulatory molecules expressed by immune cells that act as gatekeepers of the immune response to fine tune immune activation. Most traditionally explored immune checkpoints have an inhibitory, immunosuppressive role. They dampen the activation of immune cells, especially T lymphocytes, to maintain self‐tolerance and modulate the magnitude and duration of the physiological immune response following antigen stimulation, preventing overactivation and immune‐mediated pathology. Following repeated antigen stimulation, such as during a chronic infection, the expression of inhibitory immune checkpoints is upregulated, leading to a state of cellular exhaustion in which the effector function of immune cells is compromised. While this protects against continuous immune activation, it can contribute to disease pathology and infection burden by reducing the hosts' ability to mount an effective immune response and allowing pathogens to evade immune detection.

T‐cell activation normally occurs following interaction of the T‐cell receptor (TCR) with a peptide: major histocompatibility complex (MHC) on an antigen‐presenting cell (APC), as well as interaction of the co‐receptor CD28 on T cells with CD80 or CD86 on APCs. Many inhibitory immune checkpoint molecules function by blocking this pathway via direct competition with these stimulatory receptors for their ligands, thereby preventing T‐cell activation. One such example is the CD28 homolog cytotoxic T‐lymphocyte‐associated protein 4 (CTLA‐4), which directly competes with the T‐cell co‐stimulatory receptor, CD28, to prevent the costimulatory signal and block T‐cell activation.^1^, ^2^ Programmed cell death 1 (PD‐1), also expressed on activated T cells, also acts as a co‐inhibitory receptor which inhibits the downstream signaling through both the TCR and co‐receptor.^3^, ^4^, ^5^, ^6^, ^7^ Other inhibitory checkpoint molecules, like lymphocyte‐activation gene 3 (LAG‐3) and T‐cell immunoglobulin and mucin‐domain containing‐3 (Tim‐3) have multiple ligands that each interact with the receptor via distinct mechanisms to exert pleiotropic effects. A description of common inhibitory immune checkpoint molecules, their ligands and function, is given in Table 1.

**TABLE 1:** Inhibitory immune checkpoint molecules.

Immune checkpoint	Cell type	Ligand	Function
Programmed cell death 1 (PD‐1)	Primarily expressed on activated T cells, also B cells, natural killer (NK) cells, some myeloid cells, monocytes, neutrophils, and dendritic cells (DCs)^8^, ^9^	Programmed death‐ligand 1 (PD‐L1) and programmed death‐ligand 2 (PD‐L2)^3^, ^10^	PD‐1 is a co‐inhibitory receptor that inhibits the positive signals stemming from MHC:TCR interaction. This decreased signaling results in reduced T‐cell activation, proliferation and effector functions, to prevent T‐cell overactivation.^3^, ^4^, ^5^, ^6^, ^7^ PD‐1 also regulates the differentiation of memory T cells,^5^, ^6^ contributes to self‐tolerance,^11^, ^12^, ^13^, ^14^ and limits immunopathology in host tissues.^15^
Cytotoxic T‐lymphocyte‐associated protein 4 (CTLA‐4)	Activated T cells and regulatory T cells (Tregs)^16^, ^17^, ^18^, ^19^	CD80, CD86^20^, ^21^	CTLA‐4 is a structural homolog of the co‐receptor CD28, and competes for binding to its ligands, CD80 and CD86, thereby preventing the costimulatory signal that CD28 binding induces.^1^, ^2^ This negative signaling inhibits T‐cell proliferation and induces immune cell tolerance.^22^, ^23^, ^24^
Lymphocyte‐activation gene 3 (LAG‐3)	Primarily expressed on activated T cells, NK cells, activated B cells, and plasmacytoid dendritic cells (pDCs),^25^, ^26^	Major histocompatibility complex (MHC) Class II, fibrinogen‐like protein 1 (FGL‐1), and galectin‐3 (Gal‐3)^27^, ^28^, ^29^, ^30^	LAG‐3 signaling inhibits T‐cell proliferation and production of cytokines and cytotoxic molecules such as perforin and granzyme B,^31^, ^32^, ^33^ and activates plasmacytoid DCs.^34^
V‐domain immunoglobulin suppressor of T cell activation (VISTA)	Predominantly expressed on myeloid cells. Also expressed on T cells and NK cells^35^	V‐Set and immunoglobulin domain containing 3 (VSIG‐3)^36^	VISTA interaction with VSIG‐3 inhibits T cell proliferation and reduces cytokine and chemokine production by T cells,^36^ as well as supporting survival of Tregs and suppressing the antigen‐presenting capacity of APCs.^37^, ^38^, ^39^
T‐cell immunoglobulin and mucin‐domain containing‐3 (Tim‐3)	Expressed on T cells (Th1 and Th17 CD4+ T cells, CD8+ T cells and Tregs), DCs, NK cells, monocytes, and macrophages^40^, ^41^, ^42^, ^43^, ^44^, ^45^	Galectin‐9 (Gal‐9), Carcinoembryonic antigen‐related cell adhesion molecule 1 (CEACAM‐1), phosphatidylserine (PtdSer), and high mobility group box 1 protein (HMBG1)^46^, ^47^, ^48^, ^49^	Tim‐3 signaling induces apoptosis of Th1 cells,^46^ clearance of apoptotic vesicles which maintains tolerance, and enhances antigen cross presentation,^50^ as well as inhibiting T‐cell activation and proliferation.^51^, ^52^
T‐cell immunoreceptor with immunoglobulin and ITIM domain (TIGIT)	T cells and NK cells^53^	CD155, CD112, and CD113^54^	TIGIT inhibits T cell and NK cell function.^54^
B and T lymphocyte attenuator (BTLA)	T cells, B cells, NK cells, and APCs^55^	Herpesvirus entry mediator (HVEM)^56^	BTLA inhibits T‐cell proliferation and cytokine production,^57^ and is involved in the induction of peripheral T‐cell tolerance.^58^
CD244	NK cells, γδ T cells, and some CD8+ T cells, basophils, monocytes, DCs, and myeloid‐derived suppressor cells (MDSC)^59^	CD48^60^	CD244 can provide either inhibitory or stimulatory signals, to inhibit or upregulate the cytotoxicity of NK cells and CD8+ T cells,^61^ as well as inhibiting DC activation.^62^

Another class of immune checkpoint molecules exert stimulatory functions upon recognition of their ligand (Table 2). Some, such as the tumor necrosis factor (TNF) receptor superfamily members—OX40, CD27, and 4‐1BB, are co‐stimulatory molecules that contribute to the generation of effector T cells.^68^ Another stimulatory immune checkpoint is the inducible co‐stimulatory molecule (ICOS). ICOS is expressed on activated T cells and may be required for the functioning of other co‐receptors such as CD28 and OX40.^67^ The complex signaling interactions between checkpoint molecules maintains a fine balance between preventing immune overactivation causing nonspecific damage, while ensuring foreign antigens are recognized and responded to.

**TABLE 2:** Stimulatory immune checkpoint molecules.

Immune checkpoint	Cell type	Ligand	Function
Inducible co‐stimulatory molecule (ICOS)	Activated T cells^63^, ^64^	ICOS‐Ligand (ICOSL)^65^	ICOS increases T‐cell proliferation and survival,^66^ and may be required for signaling via other costimulatory molecules such as OX40, CD40, and CD28.^67^
OX40	T cells (including Tregs)^68^	OX40‐Ligand (OX40L)^68^	OX40 is a co‐stimulatory molecule contributing to generation of effector T cells, enhancing NK cell effector function and B‐cell proliferation and differentiation.^68^
Glucocorticoid‐induced TNFR‐related protein (GITR)	Expressed on Tregs at high levels and at lower levels on conventional T cells^69^, ^70^	GITR‐Ligand (GITRL)^71^	GITR is heavily involved in immunological self‐tolerance.^69^
4‐1BB	T cells (including Tregs)^68^	4‐1BB‐Ligand (4‐1BBL)^68^	4‐1BB is a costimulatory molecule contributing to generation of effector T cells and enhancing NK cell effector function.^68^
CD27	T cells, NK cells, and B cells^68^	CD70^68^	CD27 is a co‐stimulatory molecule contributing to generation of effector T cells, enhancing NK cell effector function and B‐cell proliferation and differentiation.^68^

## 2 IMMUNE CHECKPOINT MOLECULES IN DISEASE

While immune checkpoints play a crucial role in preserving tolerance and maintaining a balanced level of immune activation, they can also be upregulated following chronic antigen stimulation. In this context, expression of immune checkpoints can lead to cellular “exhaustion”, and a state of T‐cell dysfunction characterized by sustained expression of multiple inhibitory immune checkpoints and poor effector function.^72^, ^73^ This reduces the ability of the immune system to respond to the chronic antigen stimulus and can contribute to disease.

T‐cell exhaustion was first recognized in the context of chronic lymphocytic choriomeningitis virus (LCMV) infection in mice^74^ and the discovery of PD‐1 as a key protein regulating exhaustion in the LCMV infection model.^75^ Subsequently, much of the field has focused on the contribution of T‐cell exhaustion to cancer. Immune cells, especially cytotoxic T cells, are crucial in the control of some but not all tumors.^76^ The tumor microenvironment (TME) commonly displays an upregulation of immune checkpoint ligands, such as PD‐L1,^77^ and a downregulation of stimulatory molecules such as MHC I.^78^ The expression of immune checkpoints can dampen anticancer immune responses, and immune checkpoint expression on CD8+ T cells has been associated with poor clinical outcome.^79^, ^80^, ^81^

### 2.1 Immune checkpoint molecules in HIV

#### 2.1.1 Immune function

T‐cell exhaustion, marked by immune checkpoint expression, has also been implicated in the pathology of several chronic infectious diseases, including human immunodeficiency virus (HIV). HIV infects CD4+ T cells and forms a chronic infection, resulting in a depletion of CD4+ T cells and severe immunodeficiency. HIV is treated by antiretroviral therapy (ART) that prevents viral replication; however, it does not eliminate virus from latently infected cells, necessitating lifelong treatment. Despite ART, chronic immune activation persists in people with HIV (PWH), leading to adverse health outcomes.^82^ Upon HIV infection, the expression of immune checkpoint molecules is upregulated. This has been most extensively demonstrated for PD‐1, whose expression is upregulated on CD4+ and CD8+ T cells^83^, ^84^, ^85^, ^86^ in PWH. The upregulation of PD‐1 occurs preferentially on HIV‐specific T cells,^83^, ^85^, ^87^ with upregulation not observed on cells specific for other chronic viruses such as cytomegalovirus (CMV),^83^, ^87^ and is more marked in tissue sites such as rectal tissue.^88^ Several other immune checkpoint molecules are also upregulated on T cells or NK cells in PWH, including Tim‐3,^89^ LAG‐3,^90^ CTLA‐4,^86^ and TIGIT,^91^, ^92^ including on HIV‐specific T cells.^93^ This increase in checkpoint inhibitors is also seen in animal models of HIV‐infection, with PD‐1 expression increasing on CD4+^94^ and CD8+ T cells,^95^, ^96^ and Tim‐3^97^ and TIGIT^91^ expression increasing on CD8+ T cells following infection of nonhuman primates (NHPs) with simian immunodeficiency virus (SIV). This upregulation of immune checkpoint inhibitors can persist on suppressive ART, as has been observed for PD‐1 and TIGIT,^91^, ^93^, ^98^ and therefore, targeting these molecules is of high interest for HIV cure strategies.

Upregulation of checkpoint proteins largely stems from the chronic exposure to HIV antigens; however, HIV can also modulate the expression of immune checkpoint inhibitors to dampen the antiviral immune response. The HIV Nef protein upregulates the levels of Tim‐3 on the surface of CD4+ T cells using a conserved dileucine motif.^99^ HIV also incorporates PD‐L1 into its virions, to reduce T follicular helper cell (Tfh) proliferation and production of IL‐21, thereby suppressing anti‐HIV immune responses.^100^

Expression of immune checkpoint proteins has also been associated with clinical outcome, such as natural control of virus replication, HIV‐related disease progression, and some comorbidities. Highly functional CD8+ T cells are a hallmark of elite controllers, individuals whose viral loads remain low in the absence of ART.^101^, ^102^, ^103^, ^104^, ^105^ CD8+ T cells of elite controllers downregulate expression of the checkpoint molecules, TIGIT, LAG‐3, and CD244, that are upregulated on individuals with progressive disease,^91^, ^106^ suggesting upregulation of these inhibitory molecules prevents the CD8+ T cells from mounting a sufficient response to control viremia. CD4+ T cells from chronic progressors also express higher levels of PD‐1 than those from elite controllers.^107^ Expression of PD‐1,^84^, ^108^, ^109^ CTLA‐4,^86^ LAG‐3,^90^ Tim‐3,^89^ and TIGIT^91^ all correlate with HIV‐related disease progression, meaning the higher the expression of the marker, the more likely observation of disease progression. Immune checkpoint protein expression may also influence other health outcomes. In ART‐naïve PWH or PWH on ART, worse cardiac function was correlated with increased expression of LAG‐3 and PD‐1 on CD4+ T cells.^110^

#### 2.1.2 HIV latency

In contrast to cancer, immune checkpoint proteins are also important in directly interacting with HIV itself, through the establishment of latency. Latent HIV infection occurs when a virus enters a CD4+ T cell and integrates into the host genome but does not complete the viral life cycle, including transcription, translation, and viral production (reviewed in^111^). Latently infected cells are therefore not visible to the immune system and can persist indefinitely, including on antiretroviral therapy (ART). Our group were one of the first to show that PD‐1 is important for the establishment and maintenance of HIV latency.^112^ Using an *in vitro* model of HIV latency, where we directly infected resting CD4+ T cells in the presence of myeloid dendritic cells, blocking PD‐1 resulted in a significantly lower frequency of latently infected cells and an increase in productively infected cells.^112^ Our group and others have also shown that in people with HIV on ART, viral DNA is more commonly detected in CD4+ T cells that express PD‐1 compared to cells that do not express PD‐1,^113^ and this enrichment is even greater in CD4+ T cells in tissue, including the gastrointestinal tract^88^ and in lymph node.^88^, ^114^ In fact, lymph node CD4+ T cells expressing PD‐1 have been shown to be the major source of replication‐competent HIV following ART treatment in suppressed individuals.^115^ PD‐1 expression is also an indicator of the size of the HIV reservoir, with PD‐1 expression on CD4+ T cells prior to ART initiation correlating with the levels of HIV DNA following 1 year of ART.^116^ The engagement of checkpoint inhibitors can therefore directly contribute to keeping the virus in a latent state. PD‐1 engagement on CD4+ T cells from PWH inhibits virus transcription and prevents HIV reactivation,^117^ and the interaction of PD‐L1 with PD‐1 and CD155 with TIGIT on HIV‐infected CD4+ T cells restricts HIV reactivation.^118^

We have also shown that latency is further upregulated in cells coexpressing multiple checkpoint inhibitors, such as PD‐1 and Tim‐3,^112^ and PD‐1, TIGIT, and LAG‐3.^119^ Similarly, in people with HIV on ART, we showed that CD4+ T cells that express both PD‐1 and CTLA‐4, compared to cells that express neither PD‐1 or CTLA‐4, carried higher levels of HIV DNA and when stimulated with a mitogen, produced lower levels of HIV RNA, as well as reduced levels of T‐cell activation and proliferation.^120^ We concluded that double‐positive cells were characterized by greater negative signaling and a reduced capacity to respond to stimulation, thereby favoring the maintenance of HIV latency. These findings contrasted with SIV‐infected rhesus macaques on ART, where virus was more common in cells that expressed CTLA‐4 but were negative for PD‐1 expression.^121^ Together, by promoting latent infection and reducing the capacity of HIV‐specific CD8+ T cells to exert antiviral effects, immune checkpoint molecules are an important focus for work aimed at developing an HIV cure.

### 2.2 Immune checkpoint molecules in other infectious diseases

#### 2.2.1 Viral infections (other than HIV)

In addition to HIV, immune checkpoints have been implicated in the pathogenesis of several other infectious diseases, including Hepatitis B virus (HBV), a DNA virus transmitted through exposure to infectious blood or body fluids that can cause chronic inflammation of the liver, leading to cirrhosis. In CD8+ T cells, PD‐1 is upregulated during acute HBV infection,^122^ and PD‐1, Tim‐3, and CTLA‐4 are all upregulated during chronic infection,^123^, ^124^, ^125^, ^126^ while PD‐1, LAG‐3, and Tim‐3 are upregulated on CD4+ T cells.^127^, ^128^ Moreover, PD‐L1, the ligand for PD‐1, is upregulated on B cells and monocytes during chronic HBV infection.^129^ Immune checkpoint upregulation is also a feature of Hepatitis C virus (HCV) infection, with PD‐1 upregulation on HCV‐specific CD8+ T cells,^130^, ^131^, ^132^, ^133^ and is also upregulated in the context of Epstein–Barr virus (EBV).^134^

Upregulation of immune checkpoint molecules in acute infections can also occur, but is less common. In mouse models, PD‐1 is upregulated following infection with highly pathogenic influenza strains (but not low pathogenicity strains) and has been associated with delayed clearance of infection.^135^ Expression of the immune checkpoint molecules PD‐1 and CTLA‐4,^136^ and LAG‐3 and Tim‐3^137^ has also been associated with severe SARS‐CoV‐2 infection. This demonstrates that even in acute infections immune “exhaustion” or dysregulation can contribute to disease phenotype.

#### 2.2.2 Bacterial and parasitic infections

In addition to viral infections, immune checkpoints have been implicated in the pathogenesis of several parasitic and bacterial diseases. The most highly studied of these is malaria, caused by infection with the parasitic protozoans from the *Plasmodium* genus. The life cycle of malaria inside the host is complex and multistaged, with an initial liver stage followed by blood stages of infection. Immunity to malaria following infection is not sterilizing, and multiple infections are required to confer protection against disease.^138^, ^139^, ^140^ Protection is lost if consistent exposure is not maintained,^141^, ^142^, ^143^ with chronic antigen exposure therefore occurring in malaria endemic regions. It is also well known that malaria results in immunosuppression of the host immune response,^144^ including reduced IFNγ production by T cells^145^ and compromised ability to mount effective immune responses against unrelated antigens, especially polysaccharide antigens.^146^ T cells expressing multiple checkpoint markers, including PD‐1 and LAG‐3,^137^, ^147^, ^148^, ^149^, ^150^ CTLA‐4,^149^, ^150^, ^151^ OX40,^151^ and Tim‐3^137^, ^149^, ^150^, ^152^, ^153^ have been shown to be upregulated during infection with malaria in humans and contribute to immunosuppression. Mice lacking PD‐1 clear infection with a strain of malaria that is normally chronic, suggesting a role for PD‐1‐mediated suppression of CD8+ T cell function in malaria control.^154^ Additional evidence for the importance of the PD‐1:PD‐L1 interaction in malaria comes from the protective effect of PD‐L2 expressed on dendritic cells in mice, where it competes with PD‐L1 for binding of PD‐1, inhibiting the interaction of PD‐1 with PD‐L1.^155^

Checkpoint inhibition has been investigated, although less extensively, in *Mycobacterium tuberculosis* (TB) infection. PD‐1 expression is upregulated on CD4+ but not CD8+ T cells in people with TB, with this expression reduced following combination antimycobacterial treatment.^156^, ^157^, ^158^ The evidence for the role of PD‐1 in TB disease is mixed. In mice not expressing PD‐1, mortality from TB infection is dramatically increased, indicating a protective role for PD‐1 against severe disease.^159^, ^160^, ^161^ In contrast, other evidence in mice suggests PD‐1 may reduce the overproduction of IFNγ by CD4+ T cells, thereby protecting against immune‐mediated pathology.^162^

The PD‐1 pathway is affected by other bacterial and parasitic diseases, with an increase in PD‐L1 on gastric epithelial cells during *Helicobacter pylori* infection,^163^ and increased PD‐1 expression on T cells in mice infected with *Leishmania donovani*.^164^ Tim‐3 has also been implicated in the pathogenesis of many tropical parasitic diseases, such as leishmaniasis, schistosomiasis, and toxoplasmosis.^165^ Immune checkpoints play a varied role in infectious disease, with their expression having the potential to both contribute to disease and dampen overactive immune responses to protect against immune‐mediated pathology. The targeting of immune checkpoint molecules to treat disease requires care to maintain this balance to prevent induction of further disease.

## 3 IMMUNE CHECKPOINT INHIBITORS

Immune checkpoint inhibitors are drugs that block the function of immune checkpoint molecules. These most commonly take the form of monoclonal antibodies that bind immune checkpoints, or their ligands, on the surface of cells, preventing the immune checkpoint: ligand interaction and blocking signaling through that immune checkpoint. Immune checkpoint blockade was first extensively investigated and approved as a cancer therapeutic, with the approval of Ipilimumab (anti‐CTLA‐4) for malignant melanoma in 2010.^166^ This FDA approval has now extended to anti‐PD‐1 antibodies (nivolumab, pembrolizumab, cemiplimab, sintilimab, camrelizumab, and dostarlimab), anti‐PD‐L1 antibodies (durvalumab, atezolizumab, and avelumab), and an anti‐LAG‐3 antibody (relatlimab) for the treatment of a variety of cancers.^167^ Antibodies against other checkpoint inhibitors, such Tim‐3^168^ and TIGIT,^169^ are being actively developed.

Immune checkpoint antibodies are fully humanized to prevent the development of antidrug responses. Most commercially available anti‐PD‐1 antibodies are expressed in an IgG4 backbone to prevent undesired effector functions, such as antibody‐dependent cellular cytotoxicity, which may contribute to adverse events.^170^ In addition to intravenous infusions of mAb, other delivery methods and non‐antibody formats of immune checkpoint inhibitors are being investigated. Different delivery methods include nanoparticles, which can be targeted to sites of interest,^171^, ^172^ or viral vector systems, such as adeno‐associated virus vectors.^173^ The use of non‐antibody checkpoint inhibitors has also been explored in early‐stage work, including peptide and small molecule inhibitors of PD‐1,^174^ and CRISPR/Cas9 knockdown of PD‐1.^175^ Efficacy of the delivery and targeting modalities will likely depend on the disease state, varying between cancer types and among distinct infectious diseases. The advantages of these novel formats are the potential to target delivery to desired organs or cell types, reduce cost and lower the likelihood of immune‐related adverse events associated with broad immune activation.

### 3.1 Immune checkpoint inhibitors in infectious diseases

**FIGURE 1:**
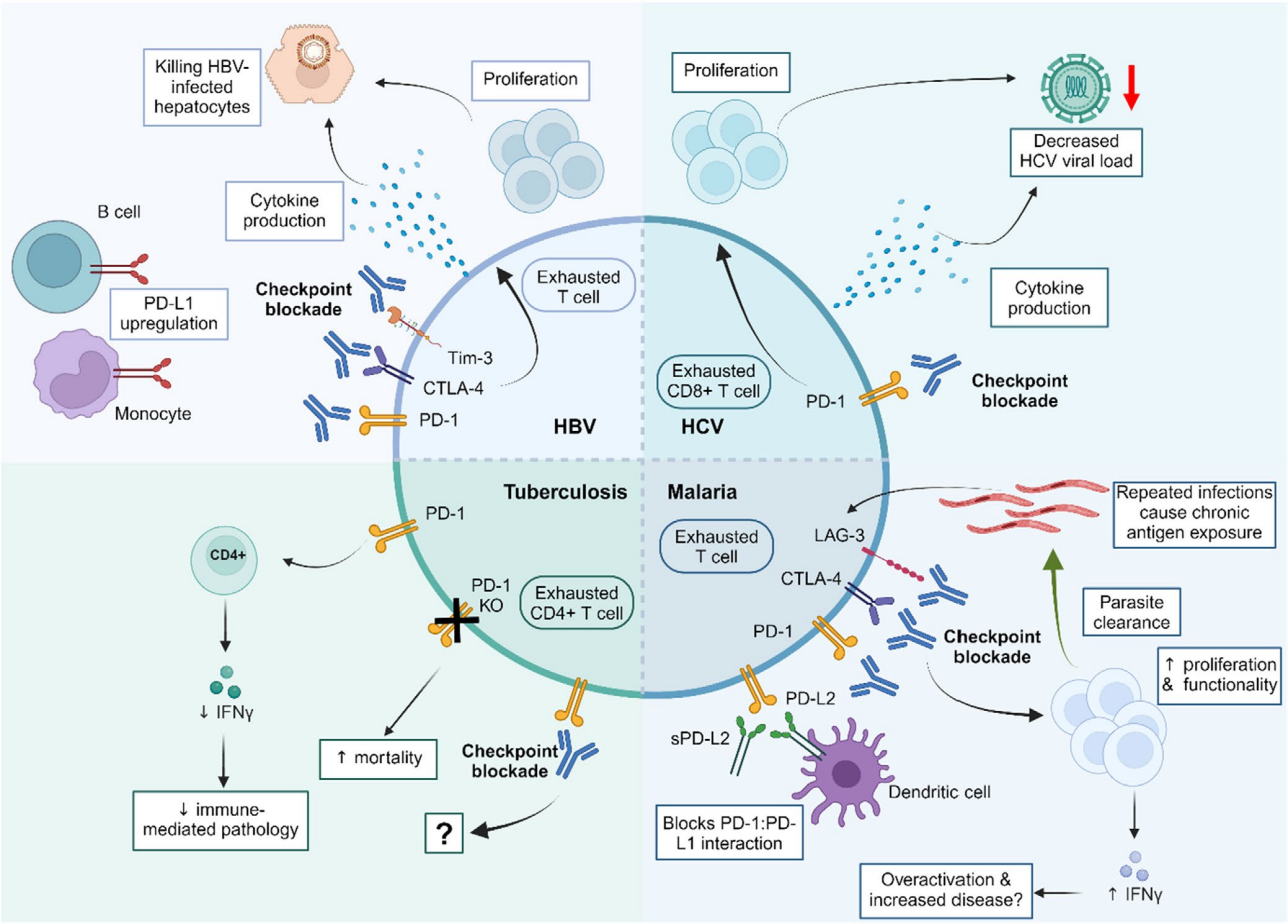
Effects of immune checkpoint blockade in different infection types. Immune checkpoint blockade has similar but distinct effects in hepatitis B virus (HBV), hepatitis C virus (HCV), malaria, and tuberculosis infection. CTLA‐4, Cytotoxic T‐lymphocyte associated protein 4; LAG‐3, Lymphocyte‐activation gene 3; PD‐1, Programmed cell death‐1; PD‐L1, Programmed cell death ligand 1; PD‐L2, Programmed cell death ligand 2; sPD‐L2, Soluble programmed cell death ligand 1; Tim‐3, T‐cell immunoglobulin and mucin‐domain containing‐3. Created with BioRender.com.

Following their successes in the cancer field, immune checkpoint blockade is being trialed as a therapeutic intervention in several chronic infectious diseases, including HBV, HCV, malaria, and HIV. Given the toxicity of immune checkpoint inhibitors, applications in infectious disease are largely only being explored in chronic infections, for which there is no cure. A summary of the effects of checkpoint blockade in these different infections is shown in Figure 1.

#### 3.1.1 Chronic viral infections

Hepatitis B virus replication is effectively inhibited using nucleos(t)ide reverse transcriptase inhibitors but can establish a long‐lived stable intermediate, called closed covalent circular DNA, which is the major reason why current antiviral treatment is unable to cure HBV. Therefore, there is now high interest in developing novel approaches to increase immune‐mediated control of virus replication that will allow people with HBV to stop lifelong antiviral treatment. Similar to HIV, chronic HBV infection is also characterized by immune dysfunction that persists on antiviral therapy.^176^ Using T cells from people with chronic HBV infection on antiviral therapy, blocking the PD‐1:PD‐L1 interaction *ex vivo* enhanced CD8+ T‐cell proliferation and cytokine production,^122^, ^124^, ^126^, ^176^, ^177^, ^178^ and CD4+ T‐cell proliferation.^179^ Blocking CTLA‐4,^123^ Tim‐3, or Tim‐3 and PD‐1 in combination, further enhanced HBV‐specific T‐cell function.^124^, ^125^ Blockade of PD‐1 exerted a greater impact than blockade of other effector pathways, largely mediated by effector memory CD8+ T cells.^124^

Infection of small animals *in vivo* with the woodchuck hepatitis virus (WHV), can establish a chronic infection, similar to HBV infection of humans. Control of WHV infectious has been reported with PD‐1 blockade.^180^ Similar findings were reported using a humanized mouse infected with HBV, whereby the administration of PD‐1 blockade enhanced viral clearance.^181^ There are now multiple clinical trials in people underway to assess the safety and efficacy of anti‐PD‐1 in chronic HBV infection to determine if anti‐PD‐1 used alone or in combination with another intervention such as a therapeutic vaccine or silencing (si)RNA may lead to a functional cure.^182^, ^183^, ^184^, ^185^, ^186^

In a Phase 1b study, virally suppressed participants with chronic HBV (*n* = 24) received either 0.1 or 0.3 mg/kg nivolumab.^187^ In this population, nivolumab was safe, and led to a mean 0.3‐log decline in hepatitis B surface antigen (HBsAg; a hallmark of persistent infection) in most participants, with sustained loss of HBsAg in one participant. In another study of anti‐PD‐1 in chronic HBV infection, there was a 0.57‐log reduction in HBsAg, including a sustained reduction in 2/15 participants,^188^ demonstrating that similar to outcomes from clinical trials in cancer, only a subset of participants appear to respond to anti‐PD‐1. One caution in the use of checkpoint inhibitors for HBV treatment is the possibility for enhanced cytotoxicity and migration of HBV‐specific T cell to the liver resulting in deranged liver function (or what is called a hepatic flare), indicating the need for careful monitoring of such participants.^189^

Prior to the advent of highly successful direct acting antivirals for hepatitis C, there was also high interest in using anti‐PD‐1 in chronic HCV infection. Although not being currently pursued given the high rates of cure following direct acting antivirals, earlier studies of anti‐PD‐1 in chronic HCV infection are informative. Using T cells from blood from people with chronic HCV infection, PD‐1 blockade *ex vivo* resulted in proliferation of HCV‐specific T cells.^131^, ^132^, ^190^ In chimpanzees chronically infected with HCV, an anti‐PD‐1 antibody reduced HCV viremia in one of three animals.^191^ A clinical trial in people with chronic HCV infection demonstrated the anti‐PD‐1 antibody (BMS‐936558) reduced HCV viral load in plasma by 0.5‐log in 5/45 participants (with a 4‐log reduction in 3 of these participants).^192^ A similar reduction in viral load was reported in a case report of a person living with HCV and cancer given pembrolizumab.^193^ There have also been several retrospective analyses of checkpoint blockade in people with HCV infection. While these datasets do not include information of the effect on the HCV viral load, they suggest safety of checkpoint blockade in this cohort.^194^, ^195^, ^196^

#### 3.1.2 Malaria

To date, the impact of checkpoint blockade for malaria has only been evaluated in mouse models. Administration of anti‐PD‐L1 and anti‐LAG‐3 monoclonal antibodies accelerated parasite clearance and increased the number and function of malaria‐specific T cells.^148^ A peptide PD‐1 antagonist also enhanced survival in mice infected with a lethal malaria strain.^197^ Given that PD‐L2 can compete with PD‐L1 to interact with PD‐1, administration of a soluble form of PD‐L2 has been investigated for immune checkpoint blockade, and when soluble PD‐2 was given to mice in a lethal malaria model survival improved.^155^ Antibodies against Tim‐3,^152^ CTLA‐4,^198^ and BTLA^199^ all increased clearance of parasitemia in mice, and enhanced signaling through OX40 (a stimulatory checkpoint molecule).^200^ Conversely, other studies have shown that blockade of PD‐1 and CTLA‐4 enhanced disease in some malaria models, due to T‐cell overactivation and excessive IFNγ.^200^, ^201^ Checkpoint blockade may have utility in the treatment of severe malaria but must be balanced against the potential for excessive immune stimulation and adverse events.

### 3.2 Immune checkpoint inhibitors in HIV

#### 3.2.1 Immune checkpoint impact on immune function

Checkpoint blockade exerts immunomodulatory effects, depending on whether the immune checkpoint is activating or inhibitory or the antibody is an antagonist or agonist. An activating intervention for exhausted T cells is particularly desirable in the context of HIV, as restoring function to immune cells may contribute to control of infection. Enhancement of anti‐HIV immune responses following inhibitory checkpoint blockade has been demonstrated multiple times in *in vitro* assays.

##### Immune checkpoint blockade ex vivo using cells from PWH on ART

Blocking PD‐1 *in vitro* enhanced the proliferation and functionality of HIV‐specific CD8+ T cells^85^, ^202^ and CD4+ T cells^203^ from PWH. This was also seen with an antibody to PD‐L1, which led to improved CD4+^90^, ^204^, ^205^ and CD8+ T cell function.^90^, ^91^ Blockade of other checkpoint molecules was also shown to be effective, with blockade of CTLA‐4 enhancing CD4+ T‐cell function,^86^, ^206^ TIGIT blockade enhancing CD8+ T‐cell function,^91^ and blocking the Tim‐3 pathway enhancing function and proliferation of both CD4+ and CD8+ T cells.^89^, ^202^ Checkpoint blockade has also been achieved with interventions other than antibodies. For example, a LAG‐3‐Fc chimera that blocks LAG‐3 interaction with its ligand enhanced IFNγ‐expressing CD4+ and CD8+ HIV‐specific T cells.^90^ Our group have evaluated immune checkpoint blockade on both CD4+ and CD8+ T‐cell function and found that blockade of anti‐CTLA‐4, PD‐L1, Tim‐3, TIGIT, or LAG‐3 had no impact on cytokine production following HIV peptide stimulation in CD4+ T cells.^203^ We believe that this was likely a result of participants in this study having been on long‐term ART with a high degree of immune reconstitution, in contrast to previous studies.

The effects of PD‐1 blockade may also vary with T‐cell differentiation status. PD‐1 blockade can inhibit the transition of naïve T cells to an effector memory phenotype *in vitro*, suggesting PD‐1 blockade at the time of initial antigen exposure may hinder the development of a de novo T‐cell response.^207^ Blockade of the PD‐1 pathway exerts effects not only on T cells, but also on NK cells by restoring the function of CD4+ T cells to provide help to the NK cells.^208^

Our group has also been interested to determine whether synergism can be achieved with multiple antibodies to immune checkpoints to enhance HIV‐specific T cell function. Using a panel of antibodies in combination or alone, we found that antibodies targeting Tim‐3 and BTLA together with an anti‐PD‐1 antibody, increased proliferation and cytokine production of HIV‐specific CD8+ T cells.^202^ The combination of antibodies to LAG‐3, CTLA‐4, and TIGIT enhanced cytokine production of both HIV‐specific CD4+ and CD8+ T cells.^203^ Interestingly, when using a cocktail of six checkpoint blockade antibodies, we observed inhibition of HIV‐specific T cell function, demonstrating that competition is possible with multiple antibodies and may not lead to better HIV‐specific T‐cell function.

We recently evaluated the effect of activating (rather than blocking) checkpoint proteins and investigated the role of glucocorticoid‐induced tumor necrosis factor related protein (GITR) in HIV‐specific T cell function.^209^ GITR is a co‐stimulatory immune checkpoint molecule constitutively expressed on regulatory T cells (Tregs) and on activated T conventional cells.^69^, ^70^ In blood collected from PWH on suppressive ART, GITR expression was reduced in multiple activated CD4+ and CD8+ T cell subsets but was increased in Tregs. HIV‐specific CD8+ T cells expressed higher levels of GITR and PD‐1 compared to total CD8+ T cells. Following stimulation with HIV peptides and GITR‐ligand (L), we demonstrated a significant decrease in killing by HIV‐specific CD8+ T cells and an increased exhaustion profile. T‐cell receptor co‐stimulation with GITR‐L abrogated Treg suppression and induced expansion of CD4+ T conventional T cells. We therefore concluded that GITR activation contributed to an impaired HIV immune response in PWH on ART, and that GITR agonist antibodies should not be pursued for HIV cure strategies.^209^

##### Immune checkpoint blockade in preclinical animal models of HIV

Blockade of PD‐1 in rhesus macaques, either chronically infected^210^ or within 2 weeks prior to ART initiation,^211^, ^212^ led to increased proliferation and enhanced effector functions of virus‐specific CD8+ T cells. In contrast, when PD‐1 was given to SIV‐infected macaques suppressed on ART, there were no changes in T‐cell activation or the magnitude of SIV‐specific T cells, likely due to the absence of antigen exposure in this context.^213^ In support of the idea that the presence of viral antigens is important for the impact of anti‐PD‐1, when PD‐1 blockade was given to SIV‐infected rhesus macaques treated with ART, at the time of analytical treatment interruption (ATI, where ART is stopped),^214^ or 1 month following ATI,^215^ there was an increase in CD4+ T cell, CD8+ T cell and NK cell proliferation, and increased expression of cytokines and the cytotoxic effector molecules perforin and Granzyme B. However, in one of these studies, there was no increase in the magnitude of SIV‐specific T cells.^215^

Anti‐PD‐1 can also affect cells other than antigen‐specific T cells, which may explain its favorable effects in SIV infection. For example, PD‐1 blockade in SIV‐infected rhesus macaques downregulated interferon‐stimulated genes, which may reduce the SIV‐associated immune activation that is associated with disease progression, thereby contributing to improved outcomes.^211^, ^216^ PD‐1 is also expressed on B cells, and in SIV infection there is a depletion of PD‐1‐expressing activated B cells.^217^ Blockade of PD‐1 in acute SIV infection was shown to enhance anti‐HIV humoral responses, B‐cell proliferation and an increase in SIV‐Env specific antibodies with a favorable clinical outcome.^210^, ^217^

Blockade of immune checkpoints other than PD‐1 has also been evaluated in the SIV model. CTLA‐4 blockade in ART‐suppressed SIV‐infected rhesus macaques demonstrated an increase of CD8+ T‐cell degranulation (by expression of CD107A) and cytokine (IFNγ and TNFα) expression by SIV‐specific CD4+ and CD8+ T cells.^218^ However, in another study, when anti‐CTLA‐4 was administered 4 days prior to and in the weeks following SIV challenge, there was no impact on SIV‐specific immune responses.^219^ In SIV‐infected nonhuman primates on suppressive ART, the administration of anti‐PD‐1 and anti‐CTLA‐4 while on suppressive ART did not enhance the magnitude of SIV‐specific CD8+ T cells, although anti‐CTLA‐4 blockade alone and combination anti‐CTLA‐4 and anti‐PD‐1 elicited proliferation of total CD4+ and CD8+ T cells, leading to an expansion of circulating effector memory T cells.^220^ In addition in this study, there was no effect of anti‐PD‐1, anti‐CTLA‐4, or both antibodies together on time to viral rebound following cessation of ART.^220^ TIGIT blockade has been investigated in chronically infected (ART‐naïve) SIV‐infected macaques, where it moderately increased the magnitude of SIV‐Gag‐specific T cells and transiently increased proliferation as measured by Ki67 expression, although overall its impact was minimal.^221^

##### Immune checkpoint blockade in vivo in PWH on ART

There have been very few studies of immune checkpoint blockade in people with HIV that also evaluated T‐cell responses. Most studies have been small and non‐randomized and performed in the setting of cancer, so that the participants are also on other potentially immunosuppressive medications which will impact the findings. A summary of outcomes from clinical trials of PWH given checkpoint blockade is shown in Tables 3 and 4, while outcomes and safety in case/cohort studies is shown in Tables S1 and S2, respectively. In some trials, an increase in HIV‐specific CD8+^224^, ^227^, ^229^, ^230^, ^231^, ^232^, ^233^, ^234^ or CD4+^224^ T cells was observed, but this is often only seen in a subset of, and not all, participants.^224^, ^227^, ^230^, ^231^ Other studies have reported increased activation marker expression on T cells,^229^, ^235^, ^236^ but no changes in the HIV‐specific immune response.^236^, ^237^

**TABLE 3:** Clinical trials of checkpoint inhibitors in PWH with cancer.

Population characteristics	Agent	Dose	Viral response	Immune response	A/Es
PWH with cancer (*n* = 32, USA)^222^, ^223^	Pembrolizumab (anti‐PD‐1)	200 mg every 3 weeks for up to 35 weeks	Increase in unspliced HIV RNA, plasma HIV RNA, and inducible virus	Not assessed	6 Grade 3 AEs
PWH with non‐small cell lung cancer (*n* = 16, France)^223^, ^224^	Nivolumab (anti‐PD‐1)	3 mg/kg every 2 weeks	No change in plasma HIV RNA	Increase in proliferating CD8+ and CD4+ T cells in a subset of participants	1 grade 3 AE (severe pruritus, onycholysis, and pemphigoid)
PWH with advanced malignancy (*n* = 40, USA)^225^, ^226^	Nivolumab ± Ipilimumab (anti‐CTLA‐4)	Nivolumab (3 mg/kg every 2 weeks), Ipilimumab (1 mg/kg every 6 weeks)	Combined PD‐1 and CTLA‐4 blockade led to modest increase in cell associated and plasma HIV RNA, small decrease in HIV DNA	Not assessed	None reported

Abbreviation: AEs, adverse events.

**TABLE 4:** Clinical trials of checkpoint inhibitors in PWH without cancer.

Population characteristics	Agent	Dose	Viral response	Immune response	A/Es
PWH on ART (no comorbidity) (*n* = 4, USA)^227^, ^228^	Cemiplimab (anti‐PD‐1)	0.3 mg/kg at weeks 0 and 6	Increase in plasma HIV RNA and decrease in total HIV DNA in one participant	Increase in polyfunctional Gag‐specific CD8+ T cells in one participant	Two irAEs (thyroiditis and elevated AST and ALT)
PWH on ART (no comorbidity) (*n* = 31, USA)^229^	Budigalimab (anti‐PD‐1)	2 mg (*n* = 9) or 10 mg (*n* = 10), both two doses 4 weeks apart, or 10 mg, four doses 2 weeks apart (*n* = 11)	Delayed viral rebound or viral control in six of nine participants receiving four doses	Increase in Tfh, CD8+ CXCR5+ and CD4+ CCR6+ cells in participants with low viral load	No serious AEs, two irAEs (thyroiditis and hyperthyroidism, both grade 1)
PWH on ART (no comorbidity) (*n* = 6, USA)^230^	BMS‐936559 (anti‐PD‐L1)	0.3 mg/kg, single infusion	No change in plasma HIV RNA or cell‐associated RNA	Increase in HIV Gag‐specific CD8+ T cells, driven by two participants	No grade 3 or higher irAEs
PWH on ART (no comorbidity) (*n* = 6, USA) (NCT03239899)	Pembrolizumab (anti‐PD‐1)	200 mg, single treatment	Increase in plasma and CD4 HIV RNA	No change in anti‐HIV antibody levels, possible increase in expression of some cytokines in the cerebrospinal fluid	No grade 3 or higher irAEs
PWH on ART (no comorbidity) (*n* = 42, Australia) (NCT05187429)	Nivolumab (anti‐PD‐1)	0.1 mg/kg, 0.3 mg/kg, or 1 mg/kg (single dose)	–	–	None recorded, trial in progress
PWH on ART (no comorbidity) (*n* = 15, China) (NCT05129189)	ASC22 (anti‐PD‐L1)	1 mg/kg every 4 weeks	–	–	None recorded, trial in progress

Abbreviations: AEs, adverse events; irAEs, immune‐related adverse events.

We recently analyzed the HIV‐specific immune response and T‐cell subsets in three people with HIV and cancer. One participant received avelumab (anti‐PD‐L1) and the other two received both ipilimumab (anti‐CTLA‐4) and nivolumab (anti‐PD‐1).^231^ One participant who received antibodies to both CTLA‐4 and PD‐1 demonstrated a striking increase in the frequency of Gag‐specific central and effector memory CD8+ T cells producing IFNγ, TNFα, and CD107A as well as an increase in frequency of CD8+ precursor exhausted T cells (Tpex).

Overall, extensive characterization of immune parameters in PWH given checkpoint blockade remains limited. Further work is needed to increase our understanding of the mechanisms behind these differing effects and identify potential biomarkers for responsiveness to immune checkpoint blockade *in vivo*.

#### 3.2.2 Immune checkpoint impact on HIV latency and the viral reservoir

**FIGURE 2:**
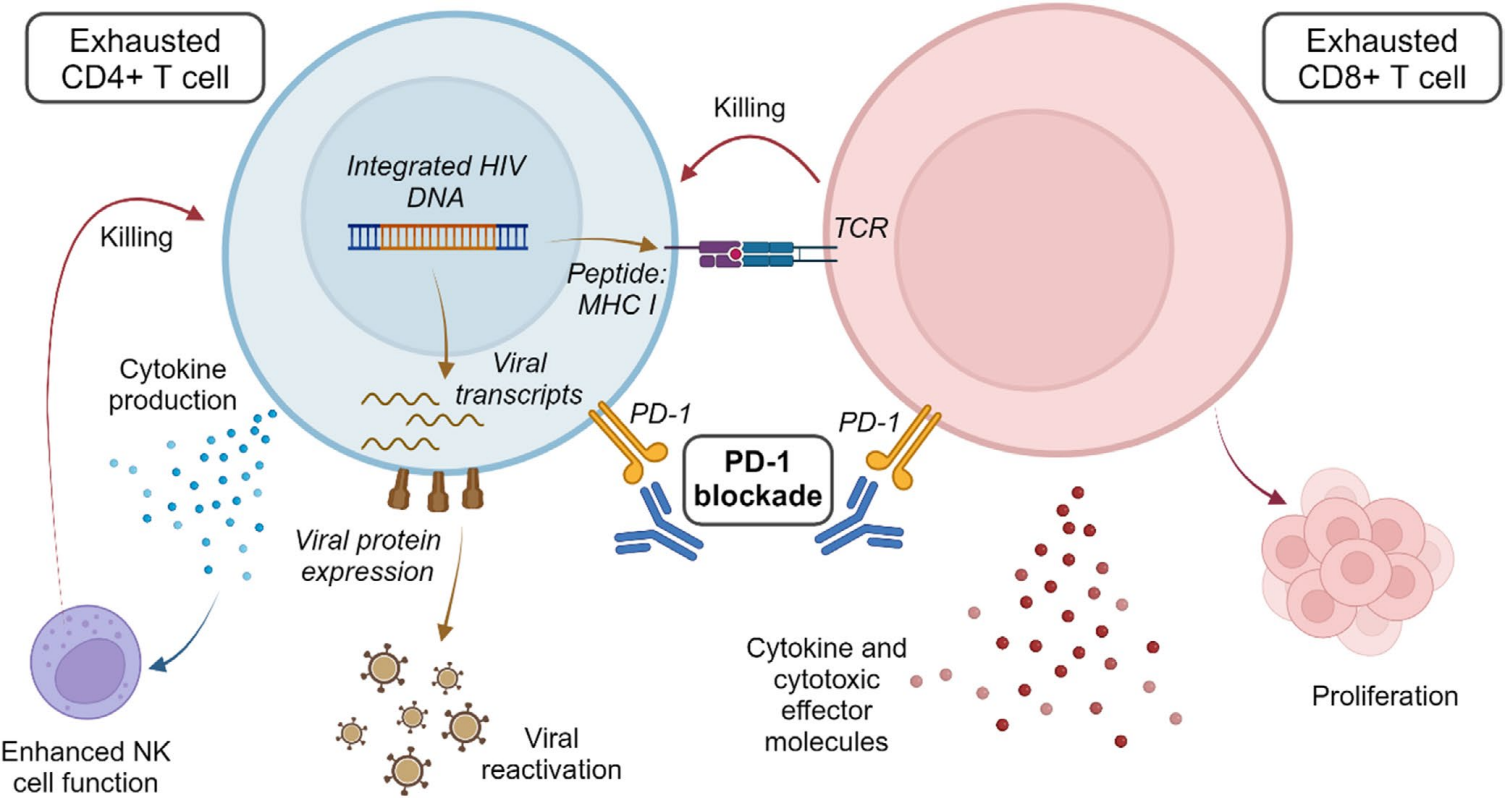
Dual effects of PD‐1 blockade on HIV infection. PD‐1 blockade activates CD4+ and CD8+ T cells, leading to production of effector molecules and proliferation. Additionally, PD‐1 blockade reactivates HIV in latently infected CD4+ T cells, leading to expression of viral proteins on the cell surface and presentation of HIV peptides by MHC I, rendering HIV‐infected cells susceptible to killing by CD8+ T or NK cells. Created with BioRender.com.

In addition to enhancement of HIV‐specific immunity, checkpoint blockade can also directly impact an infected CD4+ T cell through reactivation of latent virus (Figure 2). Given that HIV‐infected cells are enriched for the expression of checkpoint inhibitors which promote latent infection of these cells,^112^, ^119^, ^238^ we and other investigators have explored whether immune checkpoint blockade can reverse HIV latency.

Our team demonstrated that anti‐PD‐1 can reverse HIV latency in an *in vitro* model of HIV latency,^112^ using cells *ex vivo* from people with HIV on ART,^117^ and in people with HIV on ART receiving anti‐PD‐1 *in vivo*.^112^, ^117^, ^222^ In these studies, we assessed latency reversal as either an increase in cell‐associated unspliced HIV RNA, multiply spliced HIV RNA, or an increase in HIV RNA in supernatant of cultured cells, although it is important to highlight that these measures indicate different steps in the viral life cycle.^239^ An increase in unspliced HIV RNA is indicative of initiation of HIV transcription, which may not necessarily extend to production of multiply spliced RNA, expressed viral proteins or release of virions, which are required for an effective HIV‐specific immune response or virus induced cytolysis.^239^

##### Impact of immune checkpoint blockade in vitro

Our group has also been very interested to determine if inhibiting more than one immune checkpoint could further enhance latency reversal. We first investigated this hypothesis using an *in vitro* model of HIV latency whereby resting CD4+ T cells were infected in the presence of dendritic cells, a model which favors latent over productive infection and is able to distinguish between proliferating and nonproliferating‐infected cells.^240^ In this model, we found that proliferating cells expressed multiple immune checkpoint molecules at high levels.^238^ Latent infection was enriched in proliferating cells expressing PD‐1.^238^ In contrast, nonproliferating cells expressed immune checkpoint molecules at significantly lower levels, but latent infection was enriched in cells expressing PD‐1, Tim‐3, CTLA‐4, or BTLA.^238^ In the presence of an additional T‐cell‐activating stimulus, staphylococcal enterotoxin B, antibodies to CTLA‐4 and PD‐1 reversed HIV latency in proliferating and nonproliferating CD4+ T cells, respectively.^238^ In the absence of staphylococcal enterotoxin B, only the combination of antibodies to PD‐1, CTLA‐4, Tim‐3, and TIGIT reversed HIV latency.^238^ One possible mechanism behind this latency reversal is that the inhibition of immune checkpoints promoted cell cycling, thereby increasing viral transcription.^117^

##### Impact of immune checkpoint blockade on preclinical models of HIV

**FIGURE 3:**
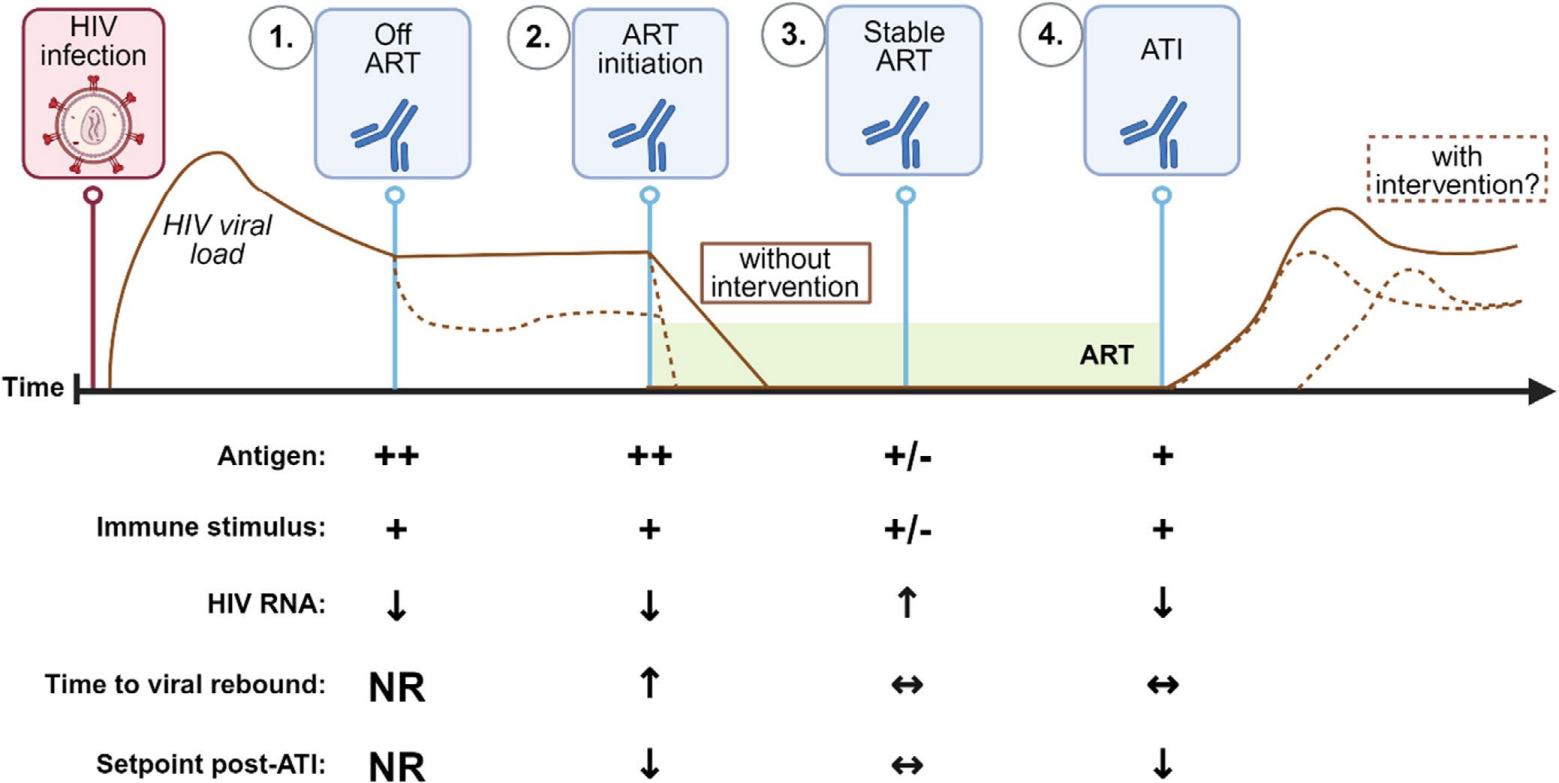
Variable effects on the HIV viral reservoir depending upon the timing of anti‐PD‐1. Antigen load at the time of anti‐PD‐1 impacts change in HIV‐specific immunity, HIV RNA, time to viral rebound, and set point post‐ATI. NR, not relevant; ↑ = increase; ↓ = decrease; ↔ = no change. Summary is based on studies in both non human primates and people with HIV. Created with BioRender.com.

There have been multiple preclinical investigations of anti‐PD‐1 in the macaque model and the outcomes differed depending on the time of administration of the antibody (Figure 3). When anti‐PD‐1 was administered to chronically SIV‐infected macaques in the absence of ART, there was a significant decline in SIV RNA and reduction in disease progression showing a very significant clinical impact.^210^ When anti‐PD‐1 was given at the time of ART initiation in SIV‐infected nonhuman primates, the control of viremia was faster than in untreated controls.^211^ In this same study, anti‐PD‐1 was administered again to the same animals following viral suppression on ART, which resulted in a reduction in the size of the inducible viral reservoir, and a delay in viral rebound and a reduced set point following ATI.^211^ Anti‐PD‐1 administered 1 month following ATI led to a transient decline in viral load in 4/7 animals following mAb administration, and a reduction of the set point viral load.^215^ In contrast, when anti‐PD‐1 was given to SIV‐infected animals on suppressive ART, there was minimal impact. For example, when anti‐PD‐1 was given in combination with a TLR7 agonist in SIV‐infected macaques on ART, there was no change in the SIV reservoir or time to viral rebound following an ATI.^213^

Our team, in collaboration with Drs. Okoye and Picker at Oregon Health Sciences University, hypothesized that the effects of anti‐PD‐1 would be greater if anti‐PD‐1 was given in the presence of viral antigen and therefore gave anti‐PD‐1 at the time of an ATI.^214^ In SIV‐infected nonhuman primates on suppressive ART, we compared the administration of an isotype control antibody to the administration of anti‐PD‐1 given either at the time of and following an ATI, or prior to and following ATI.^214^ We found that there was no difference in the time to viral rebound following ATI in all three arms, but there was a reduction in viral set point by roughly 2 logs in the nonhuman primates that received anti‐PD‐1.^214^ The administration of anti‐PD‐1 both prior to and post ATI, compared to after ATI only, did not result in any difference in viral control post ATI, indicating that administration post ATI resulted in some level of viral control.^214^

Together, these data suggest that in order to achieve ART free viral control following anti‐PD‐1 treatment, the presence of some viral antigen may be important. Consistent with this idea, the impact of immune checkpoint blockade, specifically anti‐PD‐1, on the viral reservoir in preclinical animal models varies significantly depending on whether the intervention was given to an infected macaque while viremic and off ART, at the time of initiation of ART, on stable ART or at the time of cessation of ART during an ATI (summarized in Figure 3).

The presence of antigen may also impact the effect of other immune checkpoint blockers, including anti‐CTLA‐4. The administration of anti‐CTLA‐4 to SIV‐infected ART‐treated macaques, resulted in a decrease in viral RNA in lymph nodes.^218^ In contrast, when the same antibody was given at the time of rectal challenge with SIV, there was an increase in T‐cell activation, resulting in higher plasma virus levels and faster loss of CD4+ T cells.^219^ These experiments clearly demonstrated the importance of CTLA‐4 in an effective immune response to SIV.

Dual blockade of PD‐1 and CTLA‐4 in SIV‐infected rhesus macaques on ART resulted in an increase in blips of SIV RNA in plasma, which was not observed with PD‐1 blockade alone in this model.^220^ Dual blockade of PD‐1 and CTLA‐4 also decreased the levels of total and intact SIV DNA in CD4+ T cells, and enhanced expansion of effector memory T cells.^215^ Despite these changes in the reservoir, there was no difference in time to viral rebound following ATI.^220^ There have been far fewer studies in preclinical models of the newer immune checkpoint blockers; however, when an antibody to TIGIT was administered to chronically‐infected rhesus macaques (in the absence of ART), it did not impact viral load.^221^

##### Effects of immune checkpoint blockade in PWH

The impact of checkpoint inhibitors on the HIV reservoir has been widely studied in PWH on ART receiving checkpoint blockade for cancer therapy, with the majority of reports being single case studies or observational case series (Tables S1 and S2) and more recently prospective clinical trials (Tables 3 and 4).

###### Anti PD‐1 and anti‐PD‐L1

Our team were the first to show that anti‐PD‐1 can reverse HIV latency *in vivo*.^112^, ^117^ In these studies, we showed an increase in cell associated HIV RNA, but no significant changes in plasma HIV RNA in two separate case reports.^112^, ^117^ Others have shown similar findings with increases in HIV RNA or decreases in HIV DNA in some studies,^231^, ^232^, ^234^, ^236^ but in several other studies, there appeared to be no effects on the HIV reservoir.^237^, ^241^

As part of the CITN‐12 study, we performed the largest prospective trial of anti‐PD‐1 in PWH and cancer and reported safety^223^ and impact on the HIV reservoir.^222^ We evaluated 32 participants who received pembrolizumab every 3 weeks in a virology substudy.^222^ After the first infusion of anti–PD‐1, we observed a median 1.32‐fold increase in unspliced HIV RNA and a 1.61‐fold increase in unspliced RNA:DNA ratio in sorted blood CD4+ T cells compared to baseline.^222^ We also observed a 1.65‐fold increase in plasma HIV RNA.^222^ The frequency of CD4+ T cells with inducible virus evaluated using the tat/rev limiting dilution assay was higher after six cycles compared to baseline.^222^ Phylogenetic analyses of HIV env sequences in a participant who developed low concentrations of HIV viremia after six cycles of pembrolizumab did not demonstrate clonal expansion of HIV‐infected cells.^222^ We concluded that anti–PD‐1 was able to reverse HIV latency *in vivo* and that repeated administration increased the proportion of cells with an inducible reservoir. We were unable to provide any insights on viral control as stopping ART in PWH on ART and cancer is not clinically appropriate.

There have been several small dose finding studies performed of anti‐PD‐1 in PWH on ART without cancer. These studies were primarily designed to determine safety; however, some exploratory studies of the viral reservoir were also performed. When the anti‐PD‐L1 antibody BMS‐936559 was administered to six PWH without malignancy, there were no changes in HIV plasma RNA, cell‐associated RNA, or HIV‐1 DNA.^230^ Another trial in four PWH of the anti‐PD‐1 antibody cemiplimab demonstrated a decrease in HIV DNA in one of four participants^227^; however, this trial was stopped prematurely before additional participants were enrolled due to immune‐related adverse events.

###### Anti‐CTLA‐4

The effect of anti CTLA‐4 on the HIV reservoir has also been evaluated *in vivo*, but there have been far fewer studies using this antibody, given the significant associated toxicity. Our team were the first to describe the impact of anti‐CTLA‐4 on the HIV reservoir *in vivo* in a person with HIV on ART and metastatic melanoma, in a highly cited case report.^235^ In this person, there was a striking increase in cell‐associated unspliced HIV RNA with a 19.6‐fold increase compared to baseline but no associated decline in HIV DNA following anti‐CTLA‐4.

###### Combination immune checkpoint blockade

We recently assessed the impact of combination immune checkpoint blockade on the HIV reservoir in the AMC‐095 study, in which 40 PWH with cancer received nivolumab (anti‐PD‐1) alone (*n* = 33) or nivolumab and ipilimumab (anti‐CTLA‐4; *n* = 7). In this study, there was a modest increase in cell‐associated unspliced HIV RNA following the first dose of combination therapy, although no change was observed with nivolumab monotherapy. No overall changes in measures of replication‐competent HIV were seen; however, in quantitative viral outgrowth assays from two participants who received anti‐PD‐1 and anti‐CTLA‐4 with sufficient samples available, a decrease in inducible virus was observed.^225^

#### 3.2.3 Impact of immune checkpoint blockade on viral control post‐ATI

One limitation of most studies to date of immune checkpoint blockade is that they do not include interruption of ART, as this is not feasible in a person with HIV and cancer. However, now that we have a better understanding of the safety of anti‐PD‐1, clinical trials of anti‐PD‐1 are being performed in PWH without cancer and which also include an ATI. Recently, the anti‐PD‐1 antibody budigalimab was given at the time of and following ATI, to 31 PWH without cancer. Among the nine participants who received four doses of high‐dose budigalimab, six participants displayed delayed viral rebound, and two of these participants maintained viral control (<200 copies/mL) off ART for over 29 weeks.^229^

Our team is currently enrolling participants in a study of low dose nivolumab, called NIVO‐LD (clinicaltrials.gov NCT05187429). In this study, the first phase will assess safety and receptor occupancy of low dose nivolumab in PWH without cancer in three separate dosing groups who will receive 0.1 mg/kg, 0.3 mg/kg, or 1 mg/kg nivolumab (*n* = 6/group). In the second phase of this study, participants will receive the maximum tolerated dose of nivolumab from the first phase, or a placebo, at the time of ATI (*n* = 12/group).

## 4 LIMITATIONS OF CHECKPOINT INHIBITORS

Immune checkpoint inhibitors remain a promising therapeutic for the treatment of infectious diseases, especially chronic diseases such as HIV, but several challenges will need to be met to advance these agents to the clinic. One significant challenge is the variability of the response to immune checkpoint blockade between individuals. For example, in the context of HIV, the increase in HIV RNA (consistent with latency reversal) or decrease in HIV DNA (consistent with reduction in the size of the reservoir), only occurs in some but not all participants following checkpoint blockade (Tables 3 and 4). This mirrors the experience in cancer immunotherapy, where only a proportion of participants in clinical trials are responsive to treatment.^242^ In the setting of cancer, this variability has been linked with many predictors, including the expression of PD‐L1/PD‐L2 in tumor tissue,^243^, ^244^ the presence of tumor‐infiltrating lymphocytes in the tumor microenvironment^245^ and their metabolic state,^246^ and the tumor mutational burden.^247^ Predictors of therapeutic efficacy of checkpoint blockade in PWH or other infectious diseases remains unknown. Therefore, larger clinical trials in PWH are required.

An additional consideration is the potential for adverse effects following checkpoint blockade. Of particular concern are immune‐related adverse events, driven by autoreactive T or B cells, or secondary immune activation such as changes in cytokines.^248^ Immune‐related adverse events occur in 15%–90% of participants treated with checkpoint inhibitors, with the highest incidence following CTLA‐4 blockade.^248^, ^249^ In some cases, these adverse events can be severe, with a mortality rate as high as 0.6%.^250^

Current data from PWH on ART with cancer who receive anti‐PD‐1, report a similar frequency of adverse events in this population compared to people without HIV, with this frequency not affected by CD4+ T‐cell count.^251^ The tolerance for adverse events, especially serious adverse events, is low in PWH who are otherwise well, compared to those who require checkpoint inhibitor treatment for cancer. One strategy we and others are pursuing is to reduce this risk using a lower dose and less frequent dosing of checkpoint inhibitors. The risk of adverse events in the cancer setting appears to increase with dose of checkpoint inhibitor.^252^, ^253^ By reducing this dose, as in our NIVO‐LD study (NCT05187429), we anticipate a reduction in toxicity and an acceptable safety profile for this population.

## 5 FUTURE DIRECTIONS FOR CHECKPOINT INHIBITORS IN HIV CURE

### 5.1 Combination immune checkpoint blockade with newer less toxic inhibitors

The variability of the response to checkpoint inhibitors indicates that further optimization of checkpoint inhibition strategies will likely be necessary for success in the HIV cure field. One promising avenue is the use of combination checkpoint inhibitor therapy. This may be required as administering checkpoint inhibitors can result in the compensatory upregulation of other immune checkpoints,^254^, ^255^ and HIV infection is enriched in cells expressing multiple immune checkpoints. Studies, both *in vitro* ^202^, ^203^, ^238^ and *in vivo*,^220^, ^225^, ^256^ have shown that combination checkpoint therapy can be more efficacious than single‐agent therapy. This synergism is most likely the result of distinct transcriptional signaling pathways activated by different immune checkpoints, and the expression of immune checkpoints on different cell populations, leading to stimulation of a greater number of cells when combinations are used.

**FIGURE 4:**
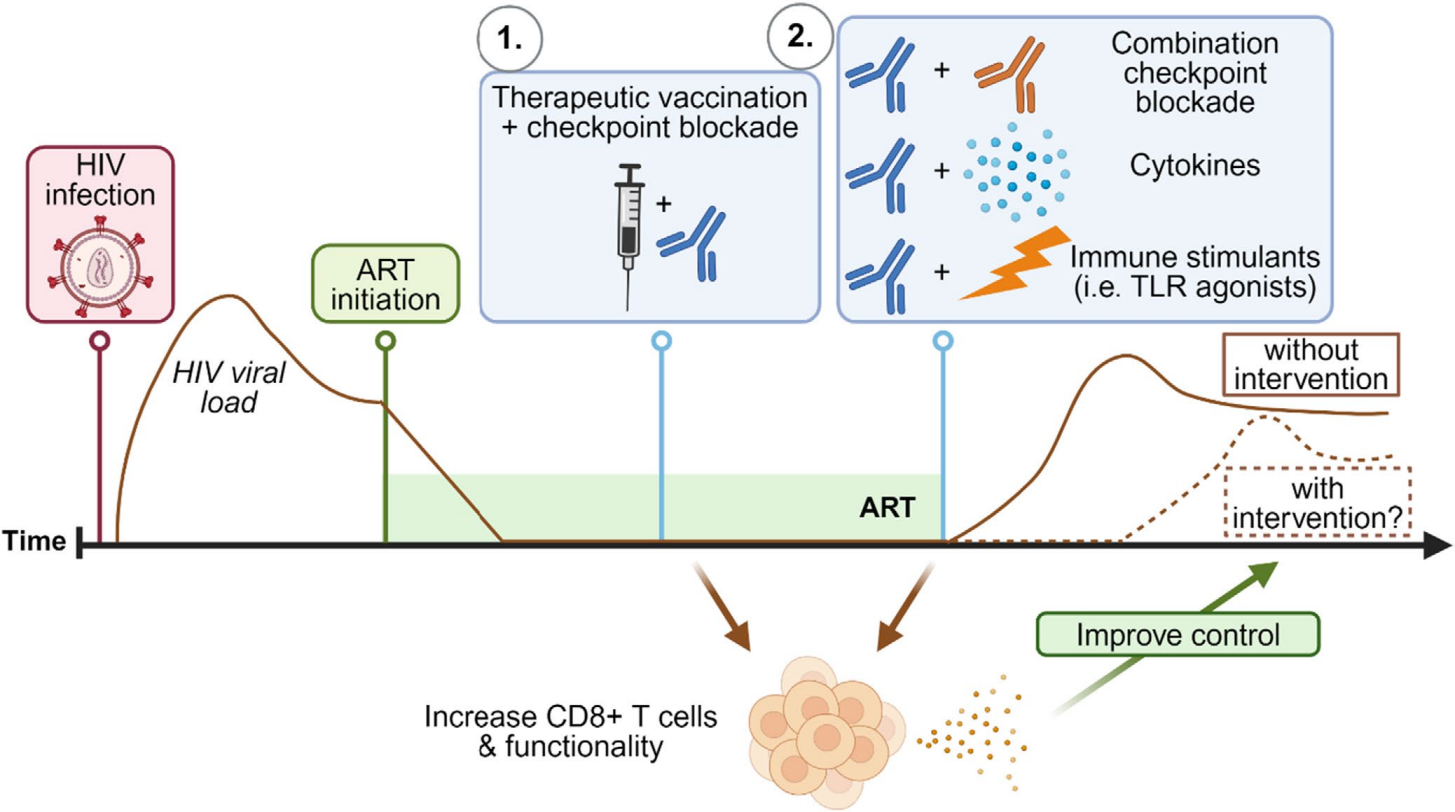
Potential applications for anti‐PD‐1 with other strategies to achieve a cure for HIV infection. (1) Anti‐PD‐1 may play a role in different strategies to achieve ART‐free viral control. Anti‐PD‐1 could be administered alongside therapeutic vaccination during ART, or (2) together with another immune checkpoint antibody such as anti‐Tim‐3, immunostimulatory cytokines or other immunomodulatory molecules at the time of ATI. These strategies aim to enhance the magnitude and functionality of HIV‐specific CD8+ T cells to improve viral control by combining checkpoint blockade with exposure to antigen. Created with BioRender.com.

The most commonly investigated combinations have been coadministration of anti‐PD‐1 and anti‐CTLA‐4 therapy; however, it is not feasible to use anti‐CTLA‐4 in PWH, given the high rates of side effects with this antibody. Other less toxic antibodies, such as anti‐LAG‐3 and anti‐Tim‐3, may be better options in people with infectious diseases. Strategies for different combination treatments with PD‐1 checkpoint blockade are given in Figure 4.

### 5.2 Cytokines and immune checkpoint blockade

Administration of the stimulatory cytokine IL‐15 or its derivatives has been extensively investigated in HIV, primarily for its ability to increase CD8+ and NK cell functionality, and its potential as a latency reversal agent.^257^ IL‐15 and an anti‐PD‐L1 antibody, avelumab, were coadministered to SIV‐infected rhesus macaques on ART, resulting in proliferation of CD8+ T cells and NK cells; however, no effect on plasma viremia following ATI was seen.^258^ *In vitro*, this same combination enhanced cytokine secretion by HIV‐specific CD8+ T cells.^259^

Another cytokine‐based combinatorial approach is co‐blockade of IL‐10 and PD‐1. IL‐10 is an inhibitory cytokine that acts on antigen‐presenting cells to reduce stimulatory receptor expression and enhance T‐cell anergy, as well as directly acting on T cells to limit proliferation and effector functions.^260^ The inhibitory effect of IL‐10 and PD‐1 are mediated through different pathways.^261^ In SIV‐infected rhesus macaques, co‐blockade of IL‐10 and PD‐1 resulted in an increase in cytokine production by antigen‐specific T cells and sustained viral control following ATI, compared to IL‐10 treatment alone.^262^

### 5.3 TLR agonists and immune checkpoint blockade

The administration of TLR7 agonists together with broadly neutralizing antibodies in nonhuman primates infected with SIV with an HIV envelope (SHIV) demonstrated control of viremia in 50% of animals following an ATI.^263^, ^264^ However, when a TLR7 agonist was administered with an anti‐PD‐1 antibody to SIV‐infected rhesus macaques, no control of viremia was observed following an ATI.^213^ Targeting mechanistically different pathways to enhance immune activation, or latency reversal agents to induce viral reactivation may lead to the greatest improvement in efficacy of checkpoint blockade.

### 5.4 Vaccination and immune checkpoint blockade

Checkpoint blockade can also be harnessed to increase the immune response to vaccination, in the setting of either prophylactic or therapeutic vaccines. In the prophylactic vaccine setting in mice, coadministering a DNA vaccine with electroporating DNA encoding PD‐L1 led to an increase in HIV‐specific CD4+ T cells and antibody responses.^265^ In nonhuman primate models, when an anti‐CTLA‐4 antibody (but not PD‐1) was administered together with a prophylactic HIV Env vaccine at both prime and boost or boost only, the generation of neutralizing antibodies was increased.^266^ PD‐1 blockade alongside a prophylactic Gag adenovirus vector vaccine at prime increased the percentage of Gag‐specific CD8+ T‐cell responses.^267^ Although the use of an immune checkpoint blocker with prophylactic vaccination is not a feasible clinical approach, these experiments are informative as they provide an understanding of how immune checkpoints can prime or boost immunity.

In the setting of therapeutic vaccination, combining an anti‐PD‐1 antibody with a single‐dose Gag T‐cell vaccine in the model system of Friend retrovirus in mice, improved CD8+ T‐cell responses and virus clearance.^268^ Enhanced CD8+ T‐cell responses have also been demonstrated following therapeutic vaccination and checkpoint blockade in the SIV model of infection, where ART‐suppressed SIV‐infected rhesus macaques were vaccinated with a DNA/modified vaccinia Ankara vaccine regimen and given an anti‐PD‐1 antibody at the time of vaccination (both prime and boost). This regimen increased the frequency of CD8+ T cells with cytotoxic functions and improved animal survival following ATI.^212^ Checkpoint blockade at the time of vaccination in the context of cellular exhaustion during chronic infection, may be a useful therapeutic tool to enhance the anti‐HIV immune response generated by vaccines.

## 6 CONCLUSIONS

Immune checkpoint molecules play an important role in the pathogenesis of many infectious diseases. During prolonged antigen stimulation caused by chronic disease, immune checkpoints can alter the immune landscape toward a suppressive state, thereby limiting the body's activity to clear infection. In this context, a promising intervention is immune checkpoint blockade, whereby monoclonal antibodies are used to block the signaling through these immune checkpoint molecules, reenergizing the immune response to achieve disease elimination. Checkpoint blockade has been investigated in the context of many infectious diseases, with the greatest amount of research focused on its potential as a therapeutic for HIV, an incurable disease affecting millions of people worldwide. Here, checkpoint blockade can exert dual effects, as both a latency reversal agent, as well as increasing the anti‐HIV immune response, which has been demonstrated through many *in vitro* and *in vivo* lines of evidence. Despite this promise, the anti‐HIV effects of checkpoint blockade have been observed in some but not all people with HIV, similar to their effects on cancer. Ongoing and future work to determine predictors of efficacy, improve delivery, understand the heterogeneity in the response, and identify effective and safe combination strategies will enable the development of checkpoint blockade as a promising treatment for individuals infected with HIV, or other infectious diseases, for which no cure or vaccine is currently available.

## CONFLICT OF INTEREST STATEMENT

SRL has received investigator‐initiated grant funding from Gilead, Merck, and ViiV Healthcare. She has participated as a paid member of scientific advisory boards to Abivax, Immunocore, Efsam, Abbvie, and Gilead.

## Supporting information


Table S1

## Data Availability

Data sharing is not applicable to this article as no new data were created or analyzed in this study.
