# Supplementary material for: Immune checkpoint inhibitors in infectious disease
Source: Immunol Rev. 2024 Sep 9;328(1):350–71. doi: 10.1111/imr.13388 (PMC11659942; doi:10.1111/imr.13388)
Supplement: Supplementary file 1 — Table S1 [file IMR-328-350-s001.docx]

**Supplementary Tables**

Table S1: Case reports and cohort studies of checkpoint inhibitors in people with HIV (PWH) with reservoir or immune measurements

| Population characteristics | Agent | Dose | Viral response | Immune response | Adverse events |
| --- | --- | --- | --- | --- | --- |
| PWH with metastatic melanoma (n=1) (1) | Nivolumab (anti-PD-1) | 3mg/kg, single dose | Decrease in HIV RNA in CD4+ T cells, no change in HIV DNA or plasma HIV RNA | Not assessed | None reported |
| PWH with non-small cell lung cancer, n=1 (2) | Nivolumab | 7 doses | Increase in HIV DNA and no change in plasma HIV RNA | Transient increase in Gag-specific CD8+ T cells, IL-6 in plasma | Grade 1 irAE, hepatic toxicity |
| PWH with non-small cell lung cancer, n=1 (3) | Nivolumab | 15 doses every 2 weeks | Decrease in HIV DNA | Increased HIV Nef-specific CD8+ T cells and T cell activation | None reported |
| PWH with metastatic melanoma (n=1) (4) | Pembrolizumab (anti-PD-1) | 2mg/kg every 3 weeks, 12 doses | Transient reduction in HIV DNA | Increased CD8+ T cell activation and functionality of HIV-specific CD8+ T cells | No AEs |
| PWH with malignancy, n=3 (5) | Nivolumab or Pembrolizumab | Variable | No consistent change in cell-associated HIV RNA or HIV DNA | Minimal HIV-specific T cell responses observed, anti-HIV antibodies remained stable | 1 irAE (autoimmune dermatitis) |
| PWH with cancer (n=32) (6) | Nivolumab, Pembrolizumab or Cemiplimab (anti-PD-1) | 2-3mg/kg every 2-3 weeks, 2-36 cycles | Transient decreases in HIV DNA in 6 participants, including to undetectable levels for 10 months in 1 participant | Slight early increase in T cell activation, no sustained change in HIV-specific immune responses | 1 grade 4 irAE (myocarditis resulting in death) |
| PWH with progressive multifocal leukoencephalopathy or solid tumours (n=14) (7) | Pembrolizumab, Sintilimab (anti-PD-1), or Toripalimab (anti-PD-1) | Pembrolizumab (100mg), Sintilimab (100mg), or Toripalimab (240mg) every 3 weeks | Stable CD4+ T cell count, HIV RNA, total HIV DNA, and cell-associated HIV-RNA | Not assessed | 7 grade 1 AEs |
| PWH with cancer (n=3) (8) | Avelumab (anti-PD-L1) (n=1) or Ipilimumab (anti-CTLA-4) and Nivolumab (n=2) | Avelumab (10mg/kg every 2 weeks), Ipilimumab (1mg/kg every 3 weeks), Nivolumab (3mg/kg every 3 weeks) | Increase in unspliced HIV RNA in all participants, no consistent change in HIV DNA or inducible virus | Increased HIV Gag-specific T cells in 1 participant | No irAEs |
| PWH with metastatic melanoma (n=1) (9) | Ipilimumab | 3mg/kg, 4 doses 3 weeks apart | Decrease in plasma HIV RNA, no change in cell-associated HIV DNA, Increase in CD4+ T cells | Increased CD4+ T cell activation | None reported |

*NOTES: AEs = adverse events; irAEs = immune related adverse events.*

Table S2: Case reports and cohort studies of checkpoint inhibitors in PWH without reservoir or immune measurements

| Population  characteristics | Agent | Dose | Viral response | Immune response | Adverse events |
| --- | --- | --- | --- | --- | --- |
| PWH with Hodgkin Lymphoma (n=1) (10) | Nivolumab (anti-PD-1) | 3mg/kg every 2 weeks, 24 doses | Reservoir not assessed, on ART, stable plasma HIV RNA and CD4+ T cell count | Not assessed | No grade 3/4 AEs or any irAEs |
| PWH with Hodgkin Lymphoma (n=1) (11) | Nivolumab | 3mg/kg every 2 weeks, 17 doses | Stable CD4+ T cell count and plasma HIV RNA on ART | Not assessed | No irAEs |
| PWH with Hodgkin lymphoma (n=1) (12) | Nivolumab | 3mg/kg every 2 weeks for 8 months | Transient increase in plasma HIV RNA and CD4+ T cell count | Not assessed | irAE (autoimmune Diabetes mellitus) |
| PWH with Kaposi sarcoma (n=1) (13) | Nivolumab | 3mg/kg every 2 weeks for 12 doses | Not assessed | Not assessed | No AEs |
| PWH with non-Hodgkin lymphoma (n=10) (14) | Pembrolizumab (anti-PD-1) | 200mg every 3-4 weeks | Stable plasma HIV RNA and CD4+ count | Not assessed | 4 grade 2 irAEs, 2 grade 3 irAEs |
| PWH with metastatic prostate cancer (n=2) (15) | Pembrolizumab | 19-36 doses | Not assessed | Not assessed | No irAEs |
| People with HIV/HCV and advanced melanoma (n=1) (16) | Pembrolizumab | 2mg/kg, 2 doses 3 weeks apart | Stable plasma HIV RNA on ART | Not assessed | No irAEs |
| PWH with adenocarcinoma of the lung (n=1) (17) | Pembrolizumab | 3 doses | Not assessed | Not assessed | AE (severe diabetic ketoacidosis) |
| PWH with progressive multifocal leukoencephalopathy (n=1) (18) | Pembrolizumab | 2mg/kg, single dose | Not assessed | Not assessed | No AEs |
| PWH with non-small cell lung cancer (n = 21), melanoma (n = 1) and head and neck cancer (n = 1) (19) | Nivolumab or Pembrolizumab | Nivolumab (3 mg/kg every 2 weeks, n=21), Pembrolizumab (200mg every 3 weeks, n=2) | Stable CD4+ T cell count, trend of a decrease in 3 participants | Not assessed | 2 grade 3 AEs, (uveitis and confusion following nivolumab; late interstitial immunoallergic lung disease related to pembrolizumab) |
| PWH with Hodgkin lymphoma (n=17) (20) | Nivolumab or Pembrolizumab | Variable | Increase in median CD4+ T cell count | Not assessed | 3 grade 3 irAEs (hypothyroidism; autoimmune pancreatitis; pneumonitis) |
| PWH with Kaposi sarcoma (n=9) (21) | Nivolumab (n=8), Pembrolizumab (n=1) | Nivolumab (3mg/kg every two weeks), Pembrolizumab (200mg, every three weeks) | Increase in CD4+ T cell count in 7 participants | Not assessed | No grade 3/4 AEs |
| PWH with non-small-cell lung cancer (n=7) (22) | Pembrolizumab (n=5), Nivolumab (n=2) | Pembrolizumab (200mg every 3 weeks), Nivolumab (3mg/kg every 2 weeks) | Decrease in CD4+ T cell count in 1 participant (423/µl to 307/µl), increase in plasma HIV RNA from undetectable to 42 or 115 copies/ml in 2 participants | Not assessed | No grade 3/4 irAEs |
| PWH and non-small cell lung cancer (n=1) (23) | Nivolumab (first line), Pembrolizumab (second line) | Nivolumab (3mg/kg for 28 administrations), Pembrolizumab (200mg, 12 administrations) | Not assessed | Not assessed | No AEs |
| PWH with advanced cancer (n=24) (24) | Camrelizumab (anti-PD-1) | 200mg every 2 weeks, 4-26 cycles | Stable CD4+ T cell count and plasma HIV RNA | Not assessed | 2 grade 3 AEs (reactive cutaneous capillary endothelial proliferation; decreased lymphocyte count) |
| PWH with urothelial carcinoma (n=9) (25) | Camrelizumab | 200mg every 3 weeks | Not assessed | Not assessed | 2 grade 3 AEs |
| PWH with bladder cancer (n=10) (26) | Tislelizumab (anti-PD-1) | 200mg every 3 weeks | Stable plasma HIV RNA and CD4+ T cell count | Not assessed | 2 grade 3/4 AEs (anaemia; increased creatinine level) |
| PWH with Hodgkin lymphoma (n=1) (27) | Sintilimab (anti-PD-L1) | 200mg/kg every 3 weeks, 9 doses | Not assessed | Not assessed | “No severe toxicity” |
| PWH with Burkitt lymphoma (n=1) (28) | Sintilimab | 200mg every 3 weeks | Not assessed | Not assessed | No AEs |
| PWH with non-small cell lung cancer (n=1) (29) | Durvalumab (anti-PD-L1) | 10mg/kg for 4 months | Not assessed | Not assessed | No grade 3/4 irAEs |
| PWH with cancer (N=390) (30) | Anti-PD-1 or PD-L1 | Variable | Not assessed | Not assessed | 7.7% grade 3/4 irAEs |
| PWH with advanced stage cancers (n=21) (31) | Anti PD-1 or anti-PD-L1 | Variable | Stable CD4+ T cell count, stable plasma HIV RNA | Not assessed | 24% irAE, 14% grade 3 or above |
| PWH with cancer (n=17) (32) | Nivolumab (n=13), Pembrolizumab (n=3), Atezolizumab (anti-PD-L1) (n=1) | Median 10 doses of drug | Stable CD4+ T cell count, undetectable plasma HIV RNA in most participants, 1 participant with low level viremia had complete RNA suppression | Not assessed | 1grade 3 AE (pneumonitis) |
| PWH with metastatic melanoma or Merkel cell carcinoma (n=10) (33) | Pembrolizumab (n = 3), Nivolumab (n = 1), Ipilimumab (anti-CTLA-4) (n = 3) or Ipilimumab plus Nivolumab (n = 3) | Variable | Stable plasma HIV RNA | Not assessed | 2 grade 3/4 irAEs (colitis; myositis |
| PWH with Merkel cell carcinoma (n=3) (34) | Pembrolizumab (all), Ipilimumab (n=1) | Pembrolizumab (2mg/kg, every 3 weeks), Ipilimumab (50mg, single dose) | Stable CD4+ T cell count, plasma HIV RNA remained undetectable | Not assessed | No grade 3/4 AEs |
| PWH with small cell carcinoma (n=1) (35) | Nivolumab and Ipilimumab | Nivolumab (1mg/kg) and Ipilimumab (3mg/kg) every 3 weeks. After 2 doses, stopped, then restarted Nivolumab 1mg/kg then Ipilimumab 1mg/kg | Plasma HIV RNA increased from undetectable to 175 copies/ml, CD4+ T cell count increased from 294 cells/µl to 593 cells/µl | Not assessed | irAE (severe drug induced acute interstitial nephritis) |

*NOTES: AEs = adverse events; irAEs = immune related adverse events.*

**Supplementary References**

1. Evans VA, van der Sluis RM, Solomon A, Dantanarayana A, McNeil C, Garsia R, et al. Programmed cell death-1 contributes to the establishment and maintenance of HIV-1 latency. Aids. 2018;32(11):1491-7.

2. Le Garff G, Samri A, Lambert-Niclot S, Even S, Lavolé A, Cadranel J, et al. Transient HIV-specific T cells increase and inflammation in an HIV-infected patient treated with nivolumab. Aids. 2017;31(7):1048-51.

3. Guihot A, Marcelin AG, Massiani MA, Samri A, Soulié C, Autran B, et al. Drastic decrease of the HIV reservoir in a patient treated with nivolumab for lung cancer. Ann Oncol. 2018;29(2):517-8.

4. Blanch-Lombarte O, Gálvez C, Revollo B, Jiménez-Moyano E, Llibre JM, Manzano JL, et al. Enhancement of Antiviral CD8(+) T-Cell Responses and Complete Remission of Metastatic Melanoma in an HIV-1-Infected Subject Treated with Pembrolizumab. J Clin Med. 2019;8(12).

5. Scully EP, Rutishauser RL, Simoneau CR, Delagreverie H, Euler Z, Thanh C, et al. Inconsistent HIV reservoir dynamics and immune responses following anti-PD-1 therapy in cancer patients with HIV infection. Ann Oncol. 2018;29(10):2141-2.

6. Baron M, Soulié C, Lavolé A, Assoumou L, Abbar B, Fouquet B, et al. Impact of Anti PD-1 Immunotherapy on HIV Reservoir and Anti-Viral Immune Responses in People Living with HIV and Cancer. Cells. 2022;11(6).

7. Zhu X, Liu X, Wan Z, Hui J, Tao R, Peng X, et al. Safety and efficacy of PD-1 inhibitors in HIV-infected patients with severe comorbidities: a prospective observational cohort study. Chin Med J (Engl). 2023;136(22):2750-2.

8. Lau JSY, McMahon JH, Gubser C, Solomon A, Chiu CYH, Dantanarayana A, et al. The impact of immune checkpoint therapy on the latent reservoir in HIV-infected individuals with cancer on antiretroviral therapy. Aids. 2021;35(10):1631-6.

9. Wightman F, Solomon A, Kumar SS, Urriola N, Gallagher K, Hiener B, et al. Effect of ipilimumab on the HIV reservoir in an HIV-infected individual with metastatic melanoma. Aids. 2015;29(4):504-6.

10. Sandoval-Sus JD, Mogollon-Duffo F, Patel A, Visweshwar N, Laber DA, Kim R, et al. Nivolumab as salvage treatment in a patient with HIV-related relapsed/refractory Hodgkin lymphoma and liver failure with encephalopathy. J Immunother Cancer. 2017;5:49.

11. Chang E, Rivero G, Patel NR, Chiao EY, Lai S, Bajaj K, et al. HIV-related Refractory Hodgkin Lymphoma: A Case Report of Complete Response to Nivolumab. Clin Lymphoma Myeloma Leuk. 2018;18(2):e143-e6.

12. Hughes MS, Pietropaolo M, Vasudevan MM, Marcelli M, Nguyen H. Checking the Checkpoint Inhibitors: A Case of Autoimmune Diabetes After PD-1 Inhibition in a Patient with HIV. J Endocr Soc. 2020;4(12):bvaa150.

13. Cesmeci E, Guven DC, Aktas BY, Aksoy S. Case of metastatic kaposi sarcoma successfully treated with anti-PD-1 immunotherapy. J Oncol Pharm Pract. 2021;27(7):1766-9.

14. Lurain K, Ramaswami R, Mangusan R, Widell A, Ekwede I, George J, et al. Use of pembrolizumab with or without pomalidomide in HIV-associated non-Hodgkin's lymphoma. J Immunother Cancer. 2021;9(2).

15. Idossa D, Friedlander T, Paller CJ, Ryan CJ, Borno HT. Case Report: Clinical Characteristics and Outcomes of HIV Positive Patients With Metastatic Prostate Cancer Treated With Immunotherapy: A Case Series and Literature Review. Front Oncol. 2022;12:910115.

16. Davar D, Wilson M, Pruckner C, Kirkwood JM. PD-1 Blockade in Advanced Melanoma in Patients with Hepatitis C and/or HIV. Case Rep Oncol Med. 2015;2015:737389.

17. Cuenca JA, Laserna A, Reyes MP, Nates JL, Botz GH. Critical Care Admission of an HIV Patient with Diabetic Ketoacidosis Secondary to Pembrolizumab. Case Rep Crit Care. 2020;2020:8671530.

18. Chatterjee T, Roy M, Lin RC, Almoujahed MO, Ahmad S. Pembrolizumab for the treatment of Progressive Multifocal Leukoencephalopathy (PML) in a patient with AIDS: A case report and literature review. IDCases. 2022;28:e01514.

19. Spano JP, Veyri M, Gobert A, Guihot A, Perré P, Kerjouan M, et al. Immunotherapy for cancer in people living with HIV: safety with an efficacy signal from the series in real life experience. Aids. 2019;33(11):F13-f9.

20. Lurain K, Zarif TE, Ramaswami R, Nassar AH, Adib E, Abdel-Wahab N, et al. Real-World Multicenter Study of PD-1 Blockade in HIV-Associated Classical Hodgkin Lymphoma Across the United States. Clin Lymphoma Myeloma Leuk. 2024.

21. Galanina N, Goodman AM, Cohen PR, Frampton GM, Kurzrock R. Successful Treatment of HIV-Associated Kaposi Sarcoma with Immune Checkpoint Blockade. Cancer Immunol Res. 2018;6(10):1129-35.

22. Ostios-Garcia L, Faig J, Leonardi GC, Adeni AE, Subegdjo SJ, Lydon CA, et al. Safety and Efficacy of PD-1 Inhibitors Among HIV-Positive Patients With Non-Small Cell Lung Cancer. J Thorac Oncol. 2018;13(7):1037-42.

23. Guaitoli G, Barbieri F, Barbolini M, Molinaro E, Emidio KD, Borghi V, et al. Pembrolizumab rechallenge in squamous non-small-cell lung cancer and HIV-positivity: a case report. Immunotherapy. 2021;13(4):277-81.

24. Wu M, Zheng X, Zhang Y, Song J, Zhao J. Camrelizumab for cancers in patients living with HIV: one-single center experience. AIDS Res Ther. 2023;20(1):23.

25. Wu M, Pang Y, Zheng X, Zhao J. Camrelizumab as adjuvant therapy in urothelial carcinomas after radical surgery in people living with HIV. Int J STD AIDS. 2023;34(10):720-7.

26. Wu M, Zheng X, Wang X, Li X, Zhang Y, Zhao J. The Efficacy and Safety of Tislelizumab as Adjuvant Treatment for Advanced or Metastatic Bladder Cancer in People Living With HIV: A Retrospective Multi-Center Study. Cancer Control. 2023;30:10732748231173475.

27. Shi Y, Li Q, Zhang W, Nan Y, Yang T, Liang X, et al. Sintilimab as salvage treatment in an HIV patient with relapsed/refractory Hodgkin: a case report. Ann Palliat Med. 2020;9(4):2414-9.

28. Zhang R, Sun J. A case report of acquired immunodeficiency syndrome (AIDS)-related refractory Burkitt lymphoma got complete remission by multidisciplinary and multi-target combined therapy. Transl Cancer Res. 2022;11(6):1806-12.

29. Kawai S, Suzuki H, Okuma Y. Durvalumab Consolidation Treatment after Chemoradiotherapy for an HIV-Positive Patient with Locally Advanced Non-Small Cell Lung Cancer. Case Rep Oncol. 2020;13(2):747-53.

30. El Zarif T, Nassar AH, Adib E, Fitzgerald BG, Huang J, Mouhieddine TH, et al. Safety and Activity of Immune Checkpoint Inhibitors in People Living With HIV and Cancer: A Real-World Report From the Cancer Therapy Using Checkpoint Inhibitors in People Living With HIV-International (CATCH-IT) Consortium. J Clin Oncol. 2023;41(21):3712-23.

31. Shah NJ, Al-Shbool G, Blackburn M, Cook M, Belouali A, Liu SV, et al. Safety and efficacy of immune checkpoint inhibitors (ICIs) in cancer patients with HIV, hepatitis B, or hepatitis C viral infection. J Immunother Cancer. 2019;7(1):353.

32. Bari S, Muzaffar J, Chan A, Jain SR, Haider AM, Adams Curry M, et al. Outcomes of Programmed Cell Death Protein 1 (PD-1) and Programmed Death-Ligand 1(PD-L1) Inhibitor Therapy in HIV Patients with Advanced Cancer. J Oncol. 2019;2019:2989048.

33. Heppt MV, Schlaak M, Eigentler TK, Kähler KC, Kiecker F, Loquai C, et al. Checkpoint blockade for metastatic melanoma and Merkel cell carcinoma in HIV-positive patients. Ann Oncol. 2017;28(12):3104-6.

34. Park SY, Church C, Alexander NA, Shinohara MM, Paulson KG, Lewis KD, et al. Immune checkpoint inhibitor therapy in HIV-associated Merkel cell carcinoma: A case series of 3 patients. JAAD Case Rep. 2021;8:28-33.

35. Husnain M, Park W, Ramos JC, Johnson TE, Chan J, Dasari A, et al. Complete response to ipilimumab and nivolumab therapy in a patient with extensive extrapulmonary high-grade small cell carcinoma of the pancreas and HIV infection. J Immunother Cancer. 2018;6(1):66.
